# Split4Blank: Maintaining consistency while improving efficiency of loading RDF data with blank nodes

**DOI:** 10.1371/journal.pone.0217852

**Published:** 2019-06-04

**Authors:** Atsuko Yamaguchi, Yasunori Yamamoto

**Affiliations:** Database Center for Life Science (DBCLS), Research Organization of Information and Systems, Kashiwa, Chiba, Japan; Swiss Institute of Bioinformatics, SWITZERLAND

## Abstract

In life sciences, accompanied by the rapid growth of sequencing technology and the advancement of research, vast amounts of data are being generated. It is known that as the size of Resource Description Framework (RDF) datasets increases, the more efficient loading to triple stores is crucial. For example, UniProt’s RDF version contains 44 billion triples as of December 2018. PubChem also has an RDF dataset with 137 billion triples. As data sizes become extremely large, loading them to a triple store consumes time. To improve the efficiency of this task, parallel loading has been recommended for several stores. However, with parallel loading, dataset consistency must be considered if the dataset contains blank nodes. By definition, blank nodes do not have global identifiers; thus, pairs of identical blank nodes in the original dataset are recognized as different if they reside in separate files after the dataset is split for parallel loading. To address this issue, we propose the Split4Blank tool, which splits a dataset into multiple files under the condition that identical blank nodes are not separated. The proposed tool uses connected component and multiprocessor scheduling algorithms and satisfies the above condition. Furthermore, to confirm the effectiveness of the proposed approach, we applied Split4Blank to two life sciences RDF datasets. In addition, we generated synthetic RDF datasets to evaluate scalability based on the properties of various graphs, such as a scale-free and random graph.

## Introduction

Recently, partly due to the rapid advancement of experimental equipment and data analysis environments, such as high-throughput sequencers, functional magnetic resonance imaging [1], and high performance computing clusters [2, 3], data driven approaches, i.e., data-intensive science, have become increasingly popular in life sciences. In such research studies, diverse types of data are produced, e.g., genome sequences and images. To understand functions in biological phenomena, we must interpret various types of large amounts of data in an integrated manner. Several public institutions, such as National Center for Biotechnology Information (NCBI) [4], the European Bioinformatics Institute (EBI) [5], and DNA Data Bank of Japan (DDBJ) [6] store such data in publicly available databases. However, such databases typically have unique formats and access methods. Therefore, researchers must understand these formats and access methods to obtain target data. In this situation, adopting the Resource Description Framework (RDF) [7] to represent these datasets has attracted the attention of database developers and users [8, 9]. The specification of RDF has become a World Wide Web Consortium (W3C) Recommendation. In the specification, Internationalized Resource Identifier (IRI) is used for globally identifiable naming schema for target objects, such as genes and proteins. In addition, the specification recommends an explicit representation of the properties of such target objects. Therefore, researchers can easily combine different datasets and focus on analyzing datasets for their research purposes.

RDF datasets are generally stored in triple stores. Life science-related RDF datasets tend to be extremely large and the number of such sets is increasing; therefore, it is important to obtain an efficient method to load such datasets to a triple store. For example, UniProt [10] has approximately 44 billion triples and is updated monthly. In addition, PubChemRDF V1.6.1 beta [11] has more than 137 billion triples, and the RDF platform of EBI contains almost 4 billion triples. Triple stores that are capable of parallel loadings, such as Virtuoso [12] and Stardog [13], are preferable for efficient data loading.

Although we can load split files of an RDF dataset into a triple store in parallel, a significant issue arises when a dataset that includes blank nodes is separated. By definition, blank nodes do not have global identifiers, and pairs of identical blank nodes in the original file are recognized as different if they reside in separate files after the file has been split for parallel loading. Thus, we require a method to split a file into multiple files without losing the consistency of blank nodes. However, this task is nontrivial if there are triples whose subject and object are both blank nodes. For example, the RDF platform of EBI had 3157280 such triples as of December 2018. Similarly, NBDC NikkajiRDF [14] has 25857876 such triples and Allie [15] has 5541602 such triples. In addition, to increase load speed, the split files should be approximately the same size. Some implementations handle the problem of blank nodes by assigning special internal identifiers to blank nodes [16] or by using custom software that allows users to assign their own identifiers [17]. However, there is no general solution for splitting an RDF file for parallel loading to a triple store.

To address this issue, we propose a Split4Blank tool. This tool uses connected component and multiprocessor scheduling algorithm to satisfy the condition that identical blank nodes are not separated. Thus, Split4Blank makes the largest split file as small as possible and the processing time as short as possible. Furthermore, to confirm the effectiveness of the proposed approach, we applied Split4Blank to two life sciences RDF datasets. In addition, we generated synthetic RDF graphs to evaluate scalability for two types of scale-free graphs and random graphs. Split4Blank is available for download at https://github.com/acopom/split4blank.

## Materials and methods

### Preliminaries

We start with the formal definition for an *RDF graph* as follows: An *RDF triple* (*s*, *p*, *o*) is an element of (*I* ∪ *B*) × *I* × (*I* ∪ *B* ∪ *L*) where *I*, *L* and *B* are a set of IRIs, a set of literals and a set of blank nodes, which are considered pairwise disjoint. In this paper, an RDF triple is simply called a triple. For a triple (*s*, *p*, *o*), *s* is called the subject, *p* the predicate and *o* the object. An RDF graph is defined as a finite set of triples.

An RDF dataset is a finite set of {*G*} ∪ {(*iri*_*i*_, *G*_*i*_)∣*iri*_*i*_ ∈ *I*, *G*_*i*_ is an RDF graph} where *G* is an RDF graph called a default graph. A pair (*iri*_*i*_, *G*_*i*_) is called a named graph. To manage triples in an RDF dataset, a triple store which stores an RDF dataset as a mutable container is generally used. To load an RDF graph into a system of a triple store, a file representing the RDF graph in a textual format such as RDF/XML [18], Turtle [19] and N-Triples [20] is loaded with IRI as the name of the RDF graph for a named graph. However, some systems of triple stores support parallel loading for an RDF graphs with a large number of triples. Thus, the RDF graph can be loaded efficiently by splitting a set of triples of an RDF graph into smaller sets of triples, and using parallel loading.

A blank node, an element in *B*, represents indicating the existence of a thing that does not have an IRI. If an RDF graph is represented within a file, the same blank nodes appearing in different triples can be identified using the labels of blank nodes. For example, in Turtle, blank nodes can be expressed as _: followed by a blank node label as an identifier locally scoped to the RDF graph. If the labels of blank nodes are same in a file, the nodes are regarded as the identical nodes. However, due to the fact that a blank node does not have a global identifier such as IRI, as written in [21–23], the presence of blank nodes in an RDF graph may cause problems especially for distributed processing. Similarly, due to the fact that a triple store cannot generally determine whether blank nodes with the same labels in different files come from the same RDF graph, parallel loading of an RDF graph including blank nodes may cause also a problem. According to the study in [24], if blank nodes with the same labels come from the different RDF graph, the blank nodes may have to be treated as different nodes.

To avoid the problem of splitting a file of an RDF graph for parallel loading, blank nodes with the same labels should not be divided. To split a large file into smaller files such that identical blank nodes are not included in different files, we formally define the RDF split problem with blank nodes as follows. For an RDF graph *G* with *n* triples and a positive integer *m* representing the number of files, we find *m* disjoint sets *D*_1_, …, *D*_*m*_ of triples in *G* with minimum *max*_*i*_|*D*_*i*_| such that any blank node *b* ∈ *D*_*i*_ does not appear in *D*_*j*_(*i* ≠ *j*) and any triple *t* ∈ *G* appears in *D*_*i*_ for some *i*.

### Proposed method

Our proposed method for the RDF split problem primarily comprises two procedures, *SPLIT* and *COMBINE* procedures. The *SPLIT* procedure splits the triples of an input RDF graph *G* into sets of triples that are as small as possible such that the identical blank nodes are not separated into different sets. The *COMBINE* procedure combines small sets of triples into *m* sets *D*_1_, …, *D*_*m*_ such that max_*i*_ |*D*_*i*_| is small.

Algorithms 1 and 2 show the SPLIT and COMBINE procedures, respectively. For an input set *T* of triples, *SPLIT* first separates *T* into three sets *T*_*b*1_, *T*_*b*2_, and *T*_0_, where *T*_*b*2_ includes triples whose subject and object are blank nodes, *T*_*b*1_ includes triples whose subject or object are blank nodes not included in *T*_*b*2_, and *T*_0_ includes triples whose subject and object are not blank nodes. By computing connected components *V*_1_, …, *V*_*k*_ for a graph constructed by *T*_*b*2_, the triples of *T*_*b*2_ are split into *T*_1_, …, *T*_*n*_. Then, each triple *t* in *T*_*b*1_ is added to *T*_*i*_, which shares the same blank node with *t*. For each triple *t*_1_, …, *t*_*m*_ in *T*_0_, a singleton for *t*_*i*_ is then created as *T*_*k*+*i*_ ≔ {*t*_*i*_}.

**Algorithm 1**: *SPLIT* Procedure

**Input**: a set *T* of triples.

**Output**: sets *T*_1_, …, *T*_*n*_ of triples such that the same blank nodes are not separated into different sets.

**Step 0** *T*_*b*1_ ≔ ∅, *T*_*b*2_ ≔ ∅, *T*_0_ ≔ ∅.

**Step 1**: For each triple *t* = (*s*, *p*, *o*) in *T*,

   if *s* and *o* are both blank nodes, *T*_*b*2_ ≔ *T*_*b*2_ ∪ {*t*},

   elseif neither *s* nor *o* is a blank node, *T*_0_ ≔ *T*_0_ ∪ {*t*},

   otherwise *T*_*b*1_ ≔ *T*_*b*1_ ∪ {*t*}.

**Step 2**: Construct a graph *G* = (*V*, *E*) with *V* ≔ {*s*|(*s*, *p*, *o*) ∈ *T*_*b*2_} ∪ {*o*|(*s*, *p*, *o*) ∈ *T*_*b*2_} and *E* ≔ {(*s*, *o*)|(*s*, *p*, *o*) ∈ *T*_*b*2_}. Compute the connected components *V*_1_, …, *V*_*k*_ of *G*.

**Step 3**: For each triple *t* = (*s*, *p*, *o*) in *T*_*b*2_, add the triple to *T*_*i*_ such that *s* and *o* are in *V*_*i*_.

**Step 4**: For each triple *t* = (*s*, *p*, *o*) in *T*_*b*1_, if *s* or *o* is in *V*_*i*_ for some *i*(1 ≤ *i* ≤ *k*), *T*_*i*_ ≔ *T*_*i*_ ∪ {*t*}, else *T*_*k*+1_ ≔ {*t*}, *V*_*k*+1_ ≔ {*s*|*s* is a blank node} ∪ {*o*|*o* is a blank node}, and *k* ≔ *k* + 1.

**Step 5**: For each triple *t* = (*s*, *p*, *o*) in *T*_0_, *T*_*k*+1_ ≔ {*t*}, *V*_*k*+1_ ≔ ∅, and *k* ≔ *k* + 1.

**Step 6**: Output *T*_1_, …, *T*_*k*_.

We can easily prove that the sets *T*_1_, …, *T*_*n*_ of triples obtained by *SPLIT* satisfy the following propositions.

**Proposition 1** *A blank node b that appears in both T*_*i*_ *and T*_*j*_(*i* ≠ *j*) *does not exist*.

**Proof** For each blank node *b*, since *V* is split into connected components, if *b* is in *V* of Step 2, then all nodes from the triples in *T*_*b*2_ that include *b* as the subject or object would belong to the same connected component. Here the connected component is denoted by *V*_*i*_. Then, all triples in *T*_*b*2_ that include *b* are in *T*_*i*_. In addition, for each triple *t* in *T*_*b*1_ including *b*, since one subject or object is *b* and the other is not a blank node, each triple would belong to *T*_*i*_. Therefore, for any blank node *b*, all triples including *b* belong to the same set *T*_*i*_.

**Proposition 2** *For any division of T*_*i*_ *into T*_*i*1_ *and T*_*i*2_ *with T*_*i*1_ ≠ ∅ *and T*_*i*2_ ≠ ∅, *there is a blank node that appears in both T*_*i*1_ *and T*_*i*2_.

**Proof** *T*_*i*_ created at Step 5 is trivial because *T*_*i*_ is a singleton.

*T*_*i*_ newly created at Step4 is also trivial because all triples in *T*_*i*_ include the same blank node.

For *T*_*i*_ based on a connected component *V*_*i*_ at Step 3, assume there is a division of *T*_*i*_ into *T*_*i*1_ and *T*_*i*2_ with *T*_*i*1_ ≠ ∅ and *T*_*i*2_ ≠ ∅ such that a blank node that appears in both *T*_*i*1_ and *T*_*i*2_ does not exist. First, we consider the case where *T*_*i*1_ or *T*_*i*2_ is a subset of *T*_*b*1_. To simplify the proof, we assume that *T*_*i*1_ is a subset of *T*_*b*1_. Then, for each triple in *T*_*i*1_ including a blank node *b*, there should be a triple including *b* in *T*_*b*2_ because *T*_*i*_ is created at Step3. Therefore, a triple including *b* should exist in *T*_*i*2_, which is a contradiction.

If *T*_*i*1_ or *T*_*i*2_ is not a subset of *T*_*b*1_, we consider two nonempty subsets $T_{i1}^{\prime }=T_{i1}\cap T_{b2}$ and $T_{i2}^{\prime }=T_{i2}\cap T_{b2}$. Here, we assume there is no blank node appearing in both $T_{i1}^{\prime }$ and $T_{i2}^{\prime }$. We consider two blank nodes *b*1 and *b*2 with $b1\in T_{i1}^{\prime }$ and $b2\in T_{i2}^{\prime }$. From this assumption, *b*1 ≠ *b*2. $T_{i1}^{\prime }\cup T_{i2}^{\prime }$ is a connected component wherein all nodes are blank nodes; thus, there should be a path between *b*1 and *b*2. Since $b1\in T_{i1}^{\prime }$ and $b2\in T_{i2}^{\prime }$, there should be a triple *t* = (*s*, *p*, *o*) such that *s* belongs to either $T_{i1}^{\prime }$ or $T_{i2}^{\prime }$ and *o* belongs to the other. Then, if $T_{i1}^{\prime }$ includes *t*, either *s* or *o* should belong to only $T_{i2}^{\prime }$, which is a contradiction. Similarly, if $T_{i2}^{\prime }$ includes *t*, this can easily lead to a contradiction.

By Proposition 1, we see that identical blank nodes belong to the same set of triples. In addition, by Proposition 2, each *T*_*i*_ is the smallest set satisfying the condition that the identical blank nodes belong to the same set of triples.

Here, we describe the *COMBINE* procedure to make *m* files with as even sizes as possible using {*T*_1_, …, *T*_*n*_}. The *COMBINE* procedure is based on an approximation algorithm for minimum multiprocessor scheduling because the problem of combining sets of triples into *m* sets with as even size as possible can be considered a problem of assigning jobs to *m* independent processors with minimum makespan by considering the size of a set as the processing time of a job. The minimum multiprocessor scheduling problem is a well-studied NP-hard optimization problem [25–27]. To reduce processing time, we employ the longest processing time algorithm [25] which is very simple and fast but it has been shown that the ratio of an obtained makespan to the minimum makespan is less than 4/3 − 1/(3*m*), which is sufficiently close to 1.

**Algorithm 2** *COMBINE* Procedure

**Input**: Sets *T*_1_, …, *T*_*n*_ of triples, and a positive integer *m*(≤ *n*)

**Output**: *m* sets {*S*_1_, …, *S*_*m*_} of triples such that, for each *T*_*i*_, there exists *j*(< *m*) such that *T*_*i*_ ⊆ *S*_*j*_.

**Step0** For each *i*(0 ≤ *i* ≤ *m*), *S*_*i*_ ≔ ∅.

**Step1** Sort *T*_1_, …, *T*_*n*_ into $T_{1}^{\prime },\ldots ,T_{n}^{\prime }$ in decreasing order.

**Step2** For *j* from 1 to *n*,

   select *k* such that |*S*_*k*_| is the smallest among |*S*_*i*_|(0 ≤ *i* ≤ *m*), and *S*_*k*_ ≔ *S*_*k*_ ∪ *T*_*j*_.

**Step3** Output {*S*_1_, …, *S*_*m*_}.

The *SPLIT* procedure runs in O(|*T*|) time because each step requires O(|*T*|) time, while the *COMBINE* procedure runs in O(*n* log *n*) because sorting *n* items requires O(*n* log *n*) time and placing *n* items requires O(*n*) time. Here, *n* ≤ |*T*|; thus, Split4Blank runs in O(|*T*|log|*T*|) time in the worst case. However, for example, by processing triples in *T*_0_ separately from other triples, the computation time of Split4Blank can be reduced.

### Implementation and availability

Based on the algorithm described above, we developed the Split4Blank tool with Java 1.8. The source code of the tool is available at https://github.com/acopom/split4blank under the MIT license. To run the tool, Java 1.8 or later is required. The executable Java ARchive (JAR) file of Split4Blank is available at Zenodo [28] with DOI:10.5281/zenodo.2652608. To execute the tool, Java 1.8 or later is required. The file format of an RDF graph should be N-Triples.

The usage is:

% java -jar -Xmx16g -Xms16g Split4Blank.jar [targetfile] [numberOfFiles]

For example,

% java -jar -Xmx16g -Xms16g Split4Blank.jar example.nt 10

## Results

From the theoretical analysis in the previous section, we obtained hypotheses that (1) the run time of Split4Blank does not depend on the number of files, (2) the run time of Split4Blank depends on the number of files but is less than O(|*T*|log|*T*|), where *T* is the set of the triples in the original RDF graph, and (3) The RDF graph loaded using an original file and the RDF graph loaded in parallel using files split by Split4Blank are isomorphic. To demonstrate them, we conducted two types of experiments. We first applied Split4Blank to real life sciences RDF datasets. In addition, we applied Split4Blank to synthetic RDF graphs to obtain the result for various sizes of RDF graphs.

For the first type of experiment, we selected Allie [15] and NikkajiRDF [14] as real RDF datasets because they include many triples whose subject and object are both blank nodes. The features of the two datasets related to our experiment are shown in Table 1. # triples, # triples(b), and # triples(b2) represent the number of triples, the number of triples with blank nodes, and the number of triples whose subject and object are blank nodes, respectively. # nodes is the number of distinct resources in the dataset, and # blank nodes is the number of distinct blank nodes in the dataset. Allie and Nikkaji datasets used for experiment are available at ftp://ftp.dbcls.jp/allie/allie_rdf/ and ftp://ftp.biosciencedbc.jp/archive/nikkaji/, respectively. For experiments, we used a default graph of each dataset.

**Table 1:** Features of Allie and NikkajiRDF datasets.

Dataset	# triples	# triples(b)	triples(b2)	# nodes	# blank nodes
Allie	143435311	102898132	5344135	28159813	12660238
NikkajiRDF	90445172	45276720	10441166	48299880	9549560

**Fig 1:**
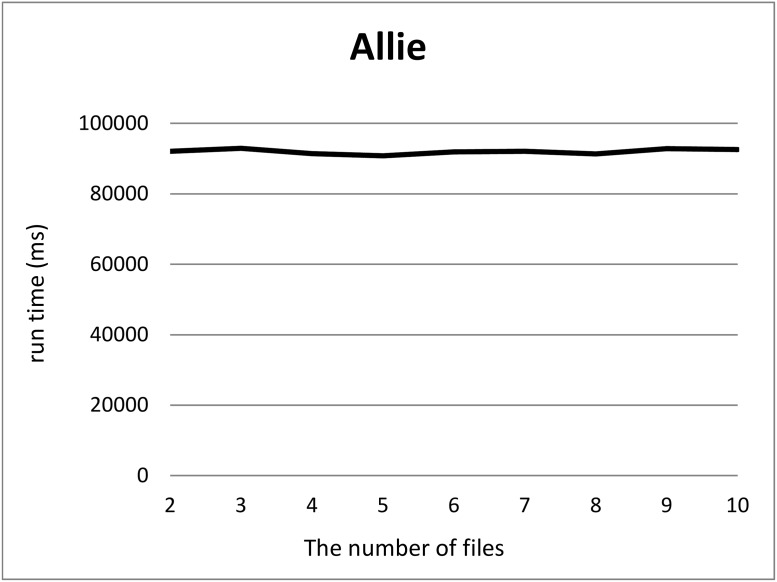
Computation time to split the Allie dataset. *x*-axis and *y*-axis correspond to the number of files from two to ten and the average of running time [ms], respectively.

**Fig 2:**
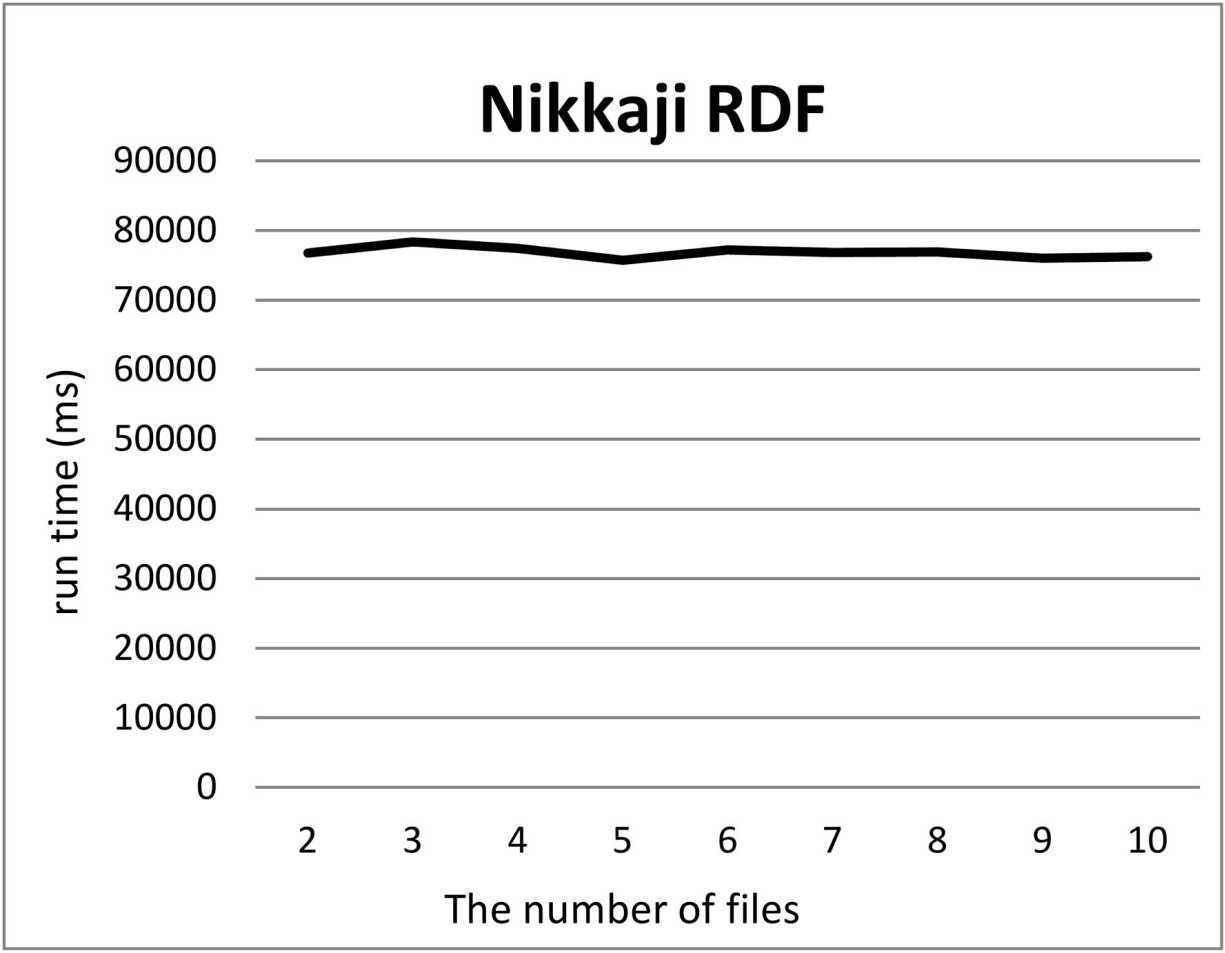
Computation time to split the Nikkaji dataset. *x*-axis and *y*-axis correspond to the number of files from two to ten and the average of running time [ms], respectively.

**Fig 3:**
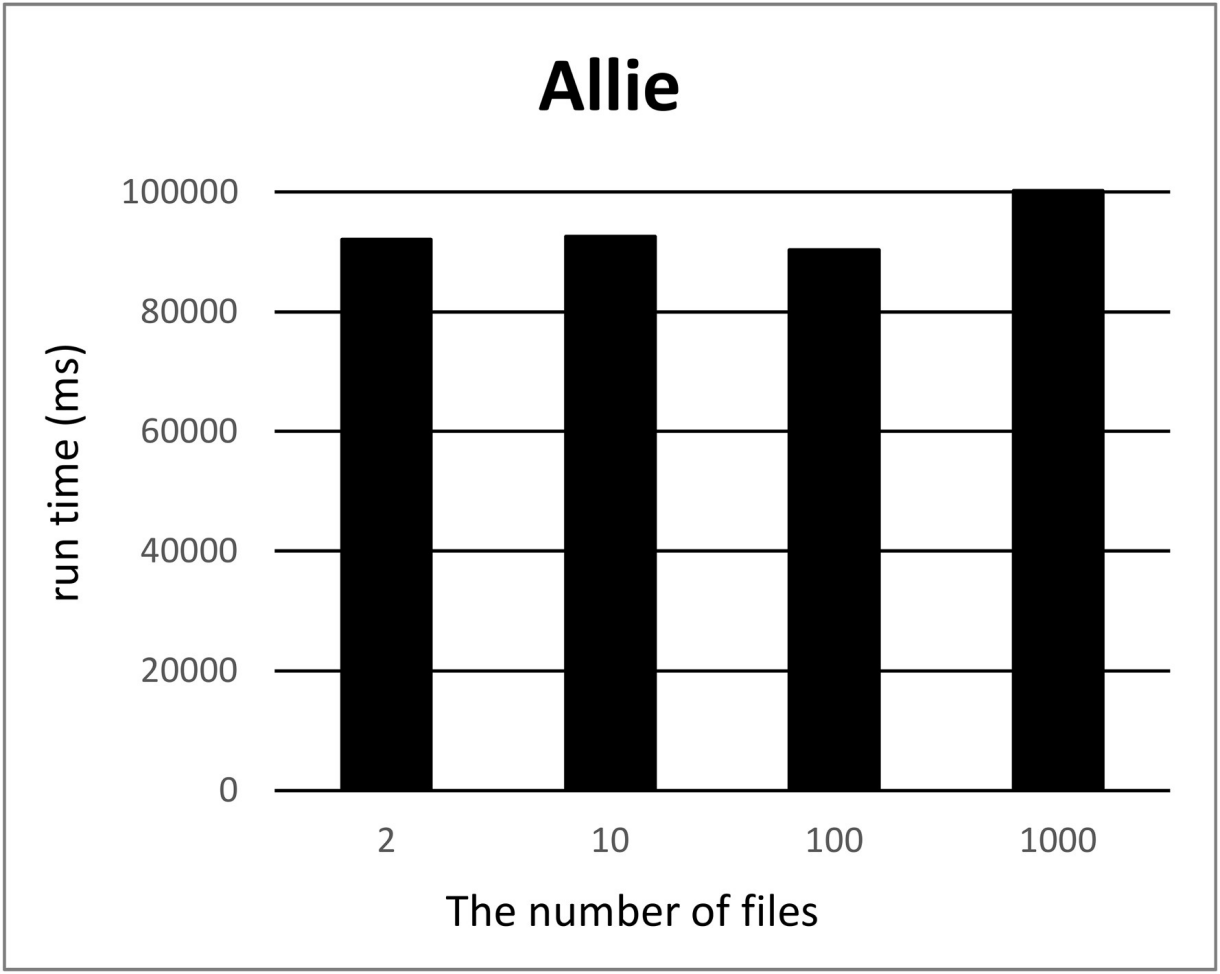
Computation time to split the Allie dataset. *x*-axis and *y*-axis correspond to the number of files (2, 10, 100, and 1000) and the average of running time [ms], respectively.

**Fig 4:**
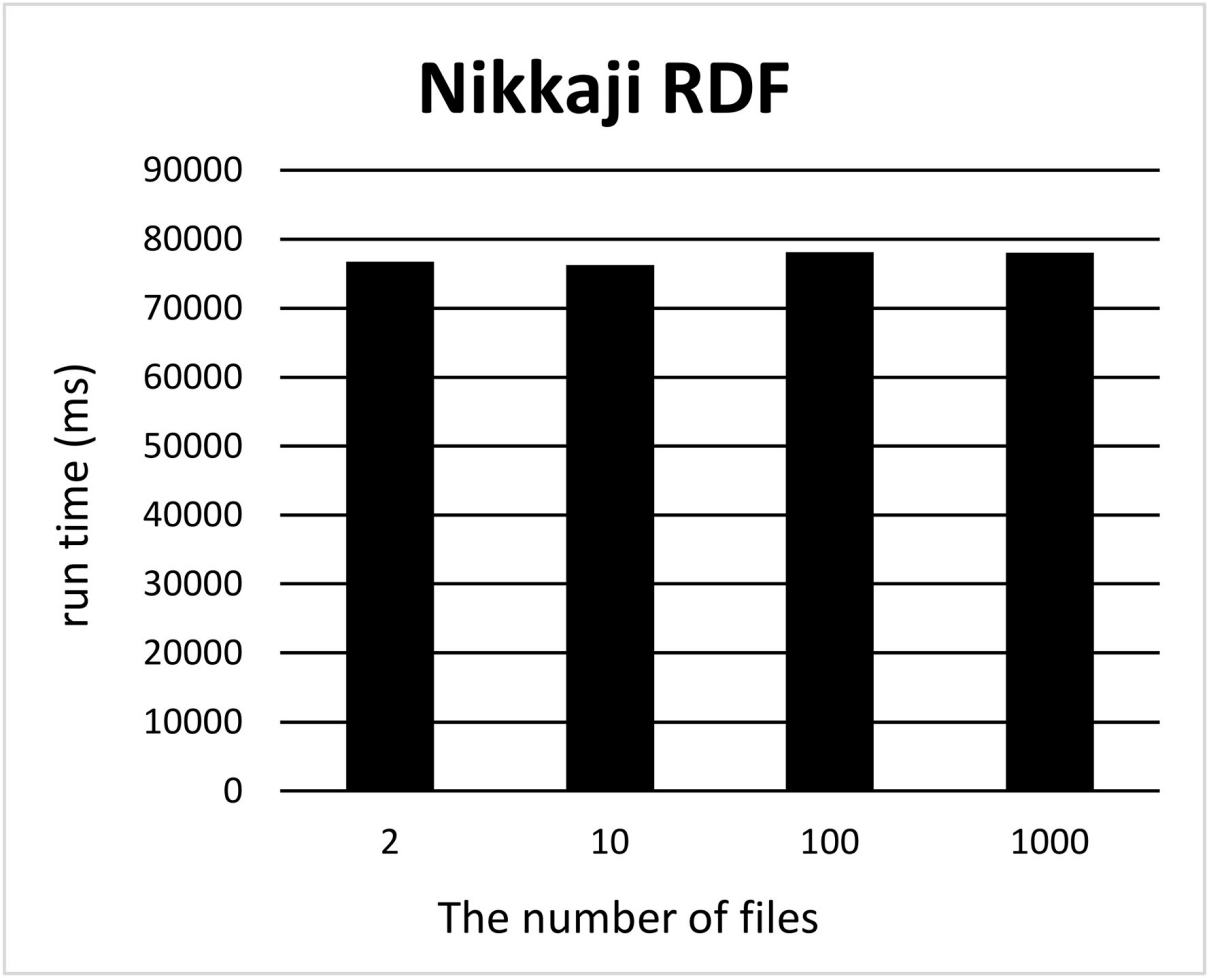
Computation time to split the Nikkaji dataset. *x*-axis and *y*-axis correspond to the number of files (2, 10, 100, and 1000) and the average of running time [ms], respectively.

We split a file of each RDF graph into *k* files where 2 ≤ *k* ≤ 10, *k* = 100 and 1000 using our tool and measured the computation time for each *k*. For each *k*, we measured the computation time 12 times, removed the minimum and maximum computation times, and took the average of ten measurements. Figs 1 and 2 show the run times for splitting triples in the Allie or NikkajiRDF datasets into *k* (2 ≤ *k* ≤ 10) sets of triples. Here, the *x*-axis and *y*-axis correspond to the number *k* of files produced by split and the run time, respectively. As can be seen, run time does not depend on the number of files. Figs 3 and 4 show the run times for splitting triples in the Allie or NikkajiRDF datasets into *k* (*k* = 2, 10, 100, and 1000) sets of triples. From the two charts, even when the number of files is 1000, the average of run time is almost the same as that when the number of files is two and we can see that the results were concordant with our hypothesis (1).

To demonstrate our hypothesis (2), we created synthetic RDF datasets of various sizes in three ways and measured the computation time required to split the datasets into two files. To create the synthetic datasets, we generated graphs of various sizes and converted them into RDF datasets by adding URIs to their nodes and edges. We employed three models to generate graphs, i.e., a random graph with *n* nodes and edge probability *p* = 0.0005, the Watts–Strogatz model with *n* nodes, initial degree *d* = 2, and rewriting probability *p* = 0.5, and the Barabasi–Albert model with *n*_0_ = 2 initial nodes, *n* additional nodes, and an additional edge parameter *e* = 30. We generated graphs using these models where the numbers *n* of nodes were 10000 to 100000.

We then assigned synthetic resource URLs (http://split4blank.dbcls.jp/experiment/node*) or blank nodes (_:bnode*) to the nodes and synthetic property URLs (http://split4blank.dbcls.jp/experiment/property*) to the edges in the generated graphs. The ratio of blank nodes to all nodes was 0.5. From the labeled graph, we generated RDF datasets for our experiments.

**Fig 5:**
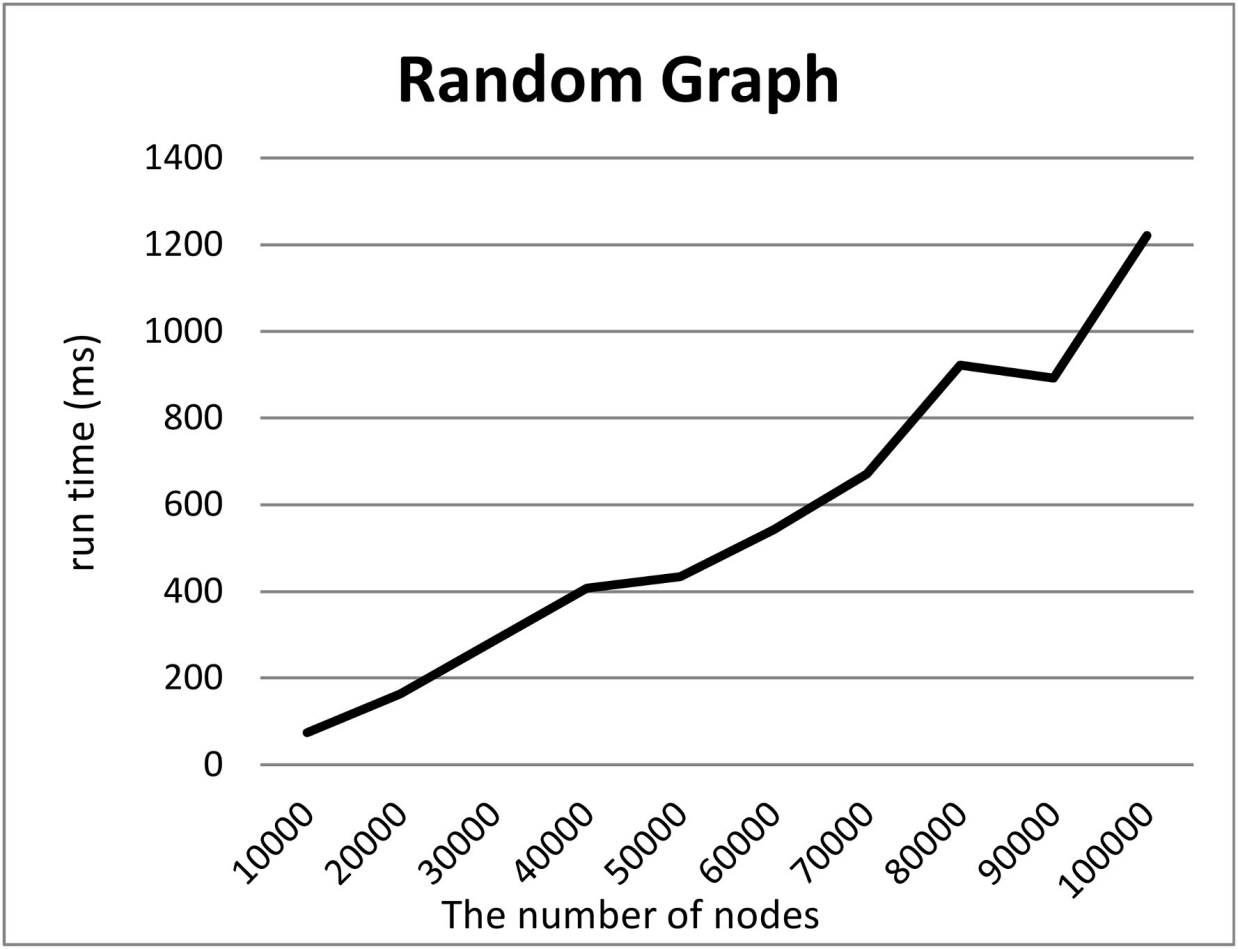
Computation time required to split the dataset generated using the random graph. *x*-axis and *y*-axis correspond to the number of nodes and the average of running time [ms].

**Fig 6:**
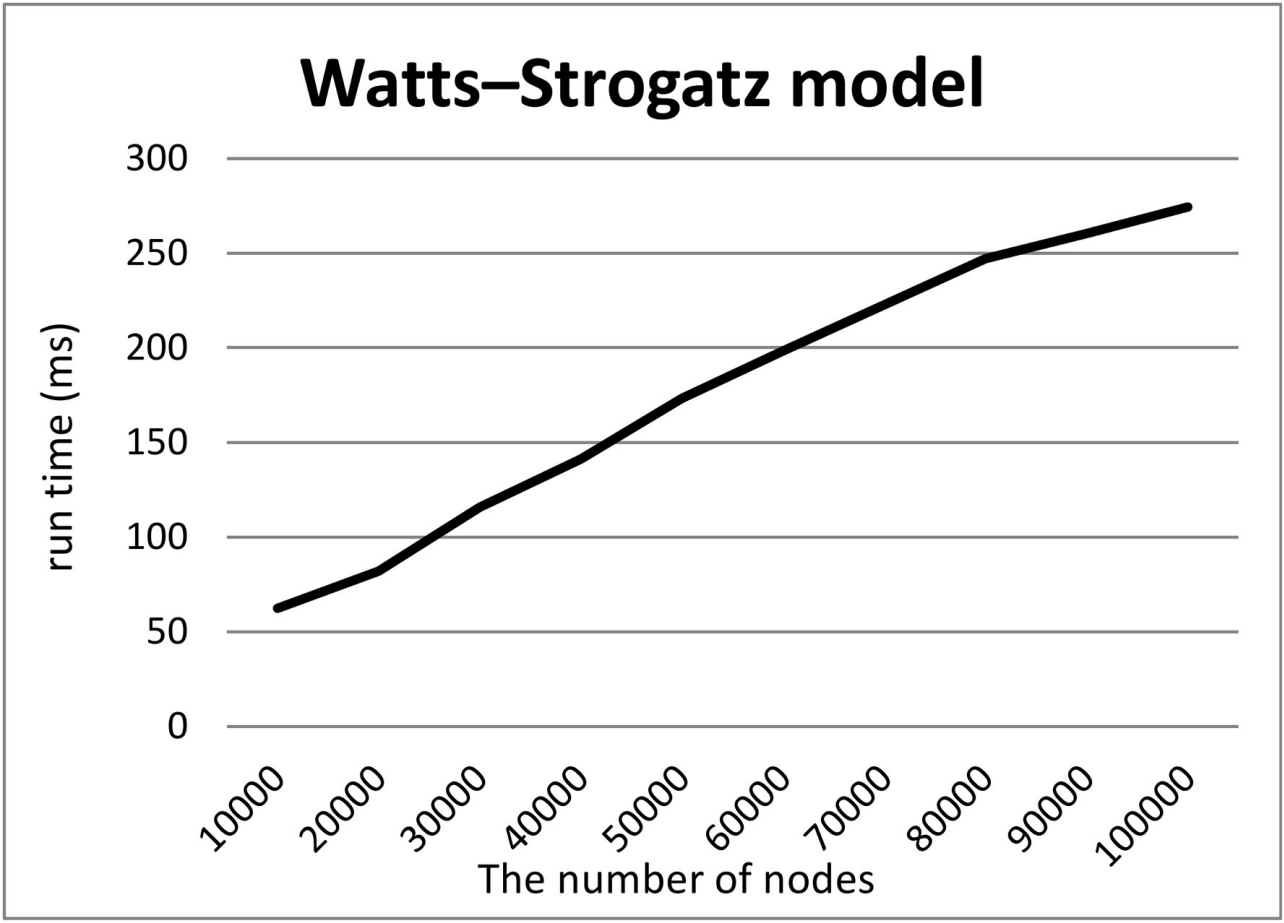
Computation time required to split the dataset generated using the Watts–Strogatz model. *x*-axis and *y*-axis correspond to the number of nodes and the average of running time [ms].

**Fig 7:**
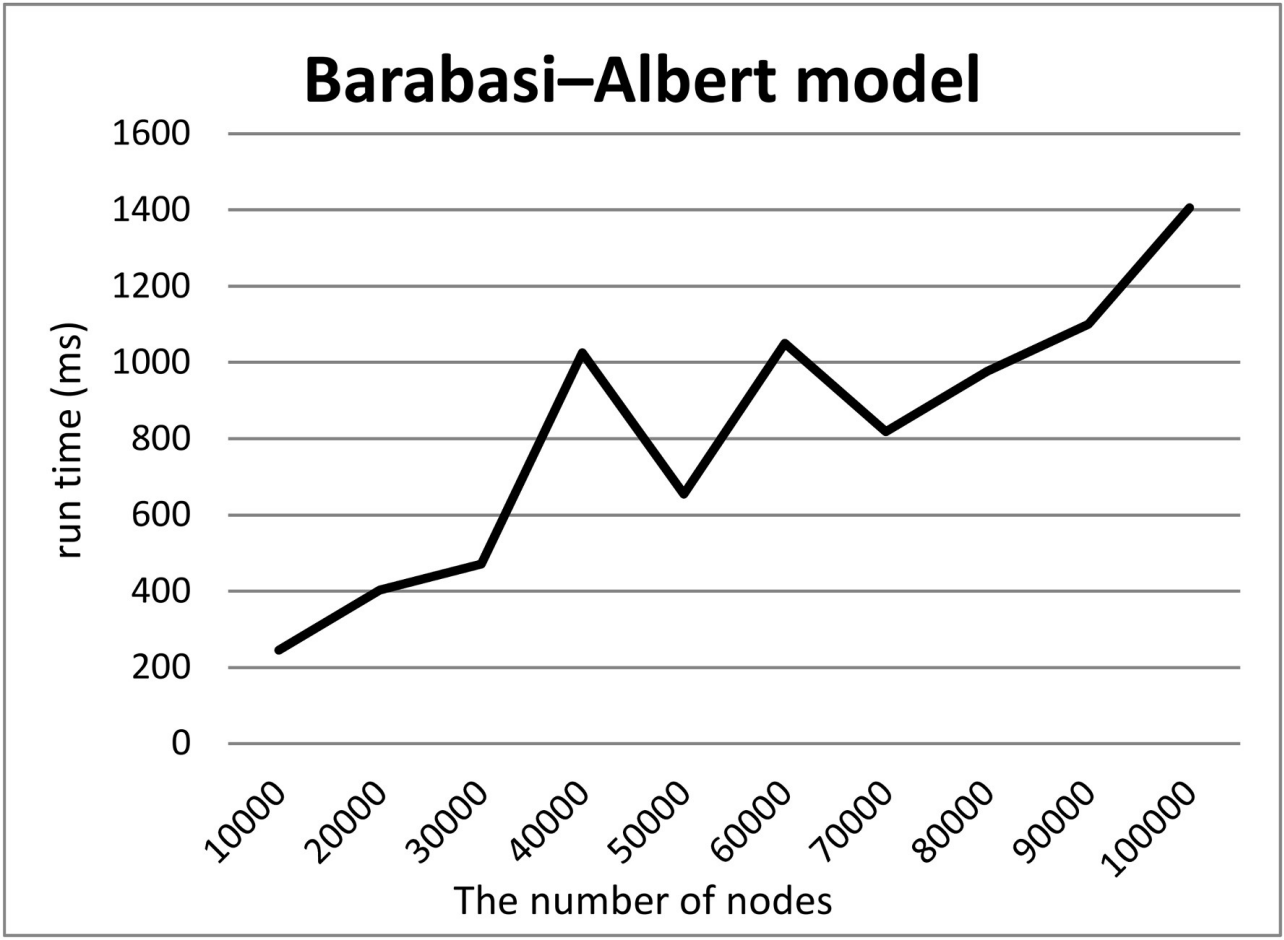
Computation time required to split the dataset generated using the Barabasi–Albert model. *x*-axis and *y*-axis correspond to the number of nodes and the average of running time [ms].

Figs 5, 6 and 7 show the computation time required to split each RDF dataset based on the random graph, Watts–Strogatz and Barabasi–Albert model, into two files. For all models, the computation times scaled linearly with the numbers of nodes although Split4Blank theoretically runs in O(|*T*|log|*T*|) time. Therefore, from a practical viewpoint, Split4Blank runs in relatively less than O(|*T*|log|*T*|) time and should be suitable for large-scale datasets.

Finally, to test the hypothesis (3), for each experiment described above, we compared two RDF graphs in a triple store when one graph is loaded using an original file and the other graph is loaded using files split by Split4Blank. As a triple store for the experiment, we used Virtuoso Open-Source Edition ver. 07.20.3229 [12]. Ideally, an isomorphism of the two graphs should be computed to test the hypothesis (3). However, the graph isomorphism problem is known to be intractable and consumes too much time for a relatively large RDF graph such as Allie and Nikkaji. Therefore, instead of computing an isomorphism, we counted the number of triples for each blank node and sorted blank nodes according to the number of triples for the two RDF graphs using the following two SPARQL queries.

Query1:

SELECT ?s (count(*) AS ?count) WHERE {

 ?s ?p ?o.

 FILTER (isBlank(?s))

}GROUP BY ?s ORDER BY DESC(?count)

Query2:

SELECT ?o (count(*) AS ?count) WHERE {

 ?s ?p ?o.

 FILTER (isBlank(?o))

}GROUP BY ?o ORDER BY DESC(?count)

Then, according to the results of these SPARQL queries, we compared the two RDF graphs and confirmed that the results are exactly the same for the two RDF graphs. Table 2 shows the top ten rows of the results of Query1 for an RDF graph loaded using an original file of Allie dataset and the RDF graph loaded using ten files split by Split4Blank. s(original) and count(original) show the result of Query1 for an RDF graph loaded using an original file. s(split) and count(split) show the result of Query1 for an RDF graph loaded using files split by Split4Blank. As can be seen, the numbers of triples for each blank node are exactly the same between the two RDF graphs. From Proposition 1, the two RDF graphs should be theoretically isomorphic. Additionally, according to the result of SPARQL queries, we can partly confirm hypothesis (3) by comparing an RDF graph using an original file and an RDF graph using files split by Split4Blank for each experiment above.

**Table 2:** The top ten rows of the results using Query1 for Allie dataset.

s (original)	count (original)	s (split)	count (split)
nodeID://b12672638	119292	nodeID://b10853750	119292
nodeID://b14420485	107999	nodeID://b8083572	107999
nodeID://b16163841	67143	nodeID://b10892334	67143
nodeID://b17913577	62619	nodeID://b10840032	62619
nodeID://b19662898	57546	nodeID://b8953824	57546
nodeID://b21404984	53633	nodeID://b11717248	53633
nodeID://b23152987	49065	nodeID://b10885370	49065
nodeID://b26646948	47950	nodeID://b11157944	47950
nodeID://b24897331	46809	nodeID://b11563642	46809
nodeID://b28394316	43445	nodeID://b8821258	43445

## Discussion

As written in [22], SPARQL engines of triple stores often offer Skolemization scheme for blank nodes. For example, values of the columns of s (original) and s (split) in Table 2, such as nodeID://b12672638 and nodeID://b10853750, are blank nodes that undergo Skolemized by Virtuoso. If there is a standardized Skolemization method of blank nodes in an RDF graph, it can also be a solution for generating split files without loss of information. However, a large number of RDF datasets including blank nodes have already been published and are circulated. Therefore, at this time, it is not realistic that all blank nodes in published RDF datasets are subjected to Skolemization.

Apache Jena Elephas (we call Elephas hereafter) [16] splits an RDF graph by generating internal identifiers from the labels of blank node present in the input file, the Job ID and the input file path. To develop an Apache Hadoop-based application that processes RDF data, the identifier is useful for building blocks for programmers However, the method used in Elephas cannot be applied in splitting an RDF dataset into multiple files to load them to any triple store in parallel, at which Split4Blank aims. A set of split files generated by Split4Blank can be loaded to any triple store that supports parallel loading.

As a limitation of our approach, if an input RDF graph includes a very large connected component of blank nodes, the file including the connected component cannot be smaller than the size of the component. However, from the practical point of view, we think this may not be a problem for almost RDF datasets because we could not find such RDF dataset in LOD although we checked many datasets in LOD for computational experiments in the Result section.

Some datasets are provided as multiple files in consideration of the above-mentioned blank node issue. However, we want to split an entire dataset into multiple files at our discretion based on machine and/or software environments. For example, the latest version (r49) of Reactome has 55 files, and we want six files in total when using a machine with six processors. In this situation, Split4Blank can be used without considering the distribution of the blank nodes.

From the scalability perspective for very large RDF datasets, our tool must consider using distributed computing, such as Apache Hadoop [29], although the experimental result demonstrates that it runs in linear time. As discussed in the Materials and Methods section, our tool primarily consists of the *SPLIT* procedure to compute connected components and the *COMBINE* procedure to solve the scheduling problem. Apache Giraph, [30] which is a graph processing framework in Apache Hadoop, includes a tool to compute connected components; thus, it may be sufficient to consider a method for solving the scheduling problem using Apache Hadoop. We employed a greedy algorithm for the problem; therefore, Map-Reduce methods for greedy algorithms [31, 32] are applicable to our tool.

## Conclusion

In this paper, we proposed a method to split an RDF dataset into several sets of triples such that identical blank nodes are stored in the same set. Furthermore, we implemented a tool and evaluated its run time in a computational experiment. In addition, from the experimental result, we conclude that the number of split files does not affect computation time and computation time scales linearly with the number of nodes.

Future work includes an investigation into the scalability of the proposed method. In this study, we used Allie and NikkajiRDF as realistic datasets and created graphs based on the random graph, Watts–Strogatz and Barabasi–Albert models. We would like to perform experiments that include more real datasets and ones based on other graph models. In particular, to adopt synthetic datasets to actual datasets, analysis of RDF datasets as graphs, such as degree distributions, is required. In addition, as discussed previously, implementing the proposed tool using Hadoop will be a focus of future work.
